# Mutation of OsKIF14.3, a Kinesin-14 Subfamily Protein, Altered Starch Metabolism and Caused Yellowish Leaf in Rice

**DOI:** 10.3390/ijms262311577

**Published:** 2025-11-29

**Authors:** Mengxue Zhang, Wenchang Jiang, Ziyu Xie, Chang Liu, Qiyu Li, Wenqiang Shen, Guanghua He, Xiaoyan Zhu

**Affiliations:** Chongqing Key Laboratory of Crop Molecular Improvement, Rice Research Institute, Academy of Agricultural Sciences, College of Agronomy and Biotechnology, Southwest University, Chongqing 400715, China; zmx20220222@163.com (M.Z.);

**Keywords:** OsKIF14.3, kinesin, OsSWEET11, excess starch accumulation, yellowish leaf mutant

## Abstract

The Kinesin superfamily members are ATP-dependent microtubule-based motor proteins that are conserved among all eukaryotic organisms and play vital roles in diverse cellular processes, such as vesicle trafficking, mitosis and meiosis, and cytoskeletal dynamics. Here, OsKIF14.3, a kinesin-14 subfamily protein, was map-based cloned and functionally analyzed. The *OsKIF14.3* gene exhibited a constitutive expression pattern. OsKIF14.3 protein localized on the microtubule and formed homodimer via the conserved Coiled Coil 1 (CC1) domain. Mutation of *OsKIF14.3* altered OsSWEET11′s subcellular location from the plasma membrane into both the plasma membrane and the cytoplasm, leading to abnormal starch metabolism, excess starch accumulation in the chloroplast, broken stroma lamella and yellowish leaves in *oskif14.3* mutant. These results enriched our understanding of the kinesin superfamily and leaf color regulation mechanism.

## 1. Introduction

As everyone knows, leaf color is a vital phenotype for plant growth and development. Owing to their ease of being observed, a large number of leaf color mutants have been discovered and those leaf color genes have been cloned and thoroughly analyzed. Most of these leaf color genes participate in photosynthesis-related pathways, for instance, *YGL3* encoding magnesium-chelatase ChlD protein, *OsCAO1* and *OsCAO2* encoding chlorophyll a oxygenase, *OsPORA* encoding NADPH: protochlorophyllide oxidoreductase A, etc., function in chlorophyll biosynthesis; *SGR* encoding *Mg^2+^-dechelatase* functions in chlorophyll degradation [1,2,3]; *OsLHCB3* encoding the light-harvesting chlorophyll a/b-binding protein functions in photosynthesis [4]. Furthermore, some of these leaf color genes were independent of photosynthesis-related pathways. For instance, *V3* and *ST1* encode the large and small subunit of ribonucleotide reductase, respectively. Both *v3* and *st1* mutants exhibited albinism phenotype in rice [5]. *ST3* encodes deoxynucleoside triphosphate triphosphohydrolase. The *rice st3* mutant exhibited white-stripe leaf phenotype during the seedling and booting stages [6]. *OsSAC3* encodes the xanthine dehydrogenase, which functions in uric acid metabolism; the *ossac3* mutant exhibited yellowish leaf with increased accumulation of starch [7]. Thus, the regulation mechanism of leaf color is fine and complicated. More genes participating in leaf color regulation should be identified and analyzed.

The kinesins constitute a functionally diverse superfamily of ATP-dependent microtubule-based motor proteins that are conserved among all eukaryotic organisms [8,9,10]. Typical kinesin consists of a motor domain, a neck-linker, a stalk and a tail [11]. Based upon homology of motor domain, the kinesin superfamily was classified into 14 subfamilies (1–14) and an orphan subfamily [12]. The kinesin-14 superfamily has numerous members in plants, such as 21 in Arabidopsis and 19 in rice. Kinesin catalyzes ATP to ADP and converts chemical energy into mechanical energy to play critical roles in mitosis, morphogenesis, and signal transduction [9,10]. Both ATK1 and ATK5 belong to the kinesin-14 subfamily and participate in chromosome segregation and spindle assembly during microsporogenesis in Arabidopsis [13,14]. KCBP belongs to the kinesin-14 subfamily, which participates in cytoskeleton assembly by integrating microtube and F-actin to regulate the morphological development of hair cells in Arabidopsis [15]. FRA1 belongs to the kinesin 4 subfamily, which regulates CMU protein level and microtube position to ensure the stability of cell wall deposition site [16]. KAC1 and KAC2 also belong to the kinesin 14 superfamily, and they mediate chloroplast movement through actin filaments [17].

In rice, among the fifty-two kinesins, only six have been cloned and analyzed. *OsPSS1* belongs to kinesin-1 subfamily and serves as a key mediator between chromosome and cytoskeleton by regulating microtubule organization and transmitting the force to nuclei to facilitate homologous chromosome pairing and synapsis in meiosis [18,19]. *GDD1*/*BC12* belongs to kinesin-4 subfamily, which functions as a dual-targeting kinesin protein and is implicated in cell-cycle progression, cellulose microfibril deposition and wall composition in rice. The *gdd1*/*bc12* mutant exhibited dwarfism and brittleness phenotype [20]. *OsKinesin-13A* is shown to be an active microtubule depolymerase, which utilizes its microtubule depolymerization activity to promote microtubule turnover and thus affects cellulose microfibril orientation and cell elongation. The mutant of OsKinesin-13A, *sar1*, displayed length reduction in grains and other organs [21]. *STD1* encodes a phragmoplast-associated kinesin-related protein and is mainly expressed in the actively dividing tissues. The STD1 protein is localized specifically to the phragmoplast midzone during telophase and cytokinesis. The *std1* mutant shows a severely dwarfed phenotype [22]. *OsKCH2* is a plant-specific kinesin-14 with an N-terminal actin-binding domain and a central motor domain flanked by two predicted coiled coils. OsKCH2 specifically decorates preprophase band microtubules in vivo and transports actin filaments along microtubules in vitro [23].

In this study, we reported the functional identification of OsKIF14.3 as a kinesin-14 superfamily member in *Oryza sativa* L. OsKIF14.3 contained a conserved motor domain and two coiled coils. OsKIF14.3 protein functioned as a homodimer and localized on the microtubule. Mutation of *OsKIF14.3* altered the subcellular location of OsSWEET11 protein, which led to abnormal starch metabolism with excess starch accumulation and yellowish leaves in the *oskif14.3* mutant.

## 2. Results

### 2.1. Phenotype Analysis of oskif14.3 Mutant

**Figure 1:**
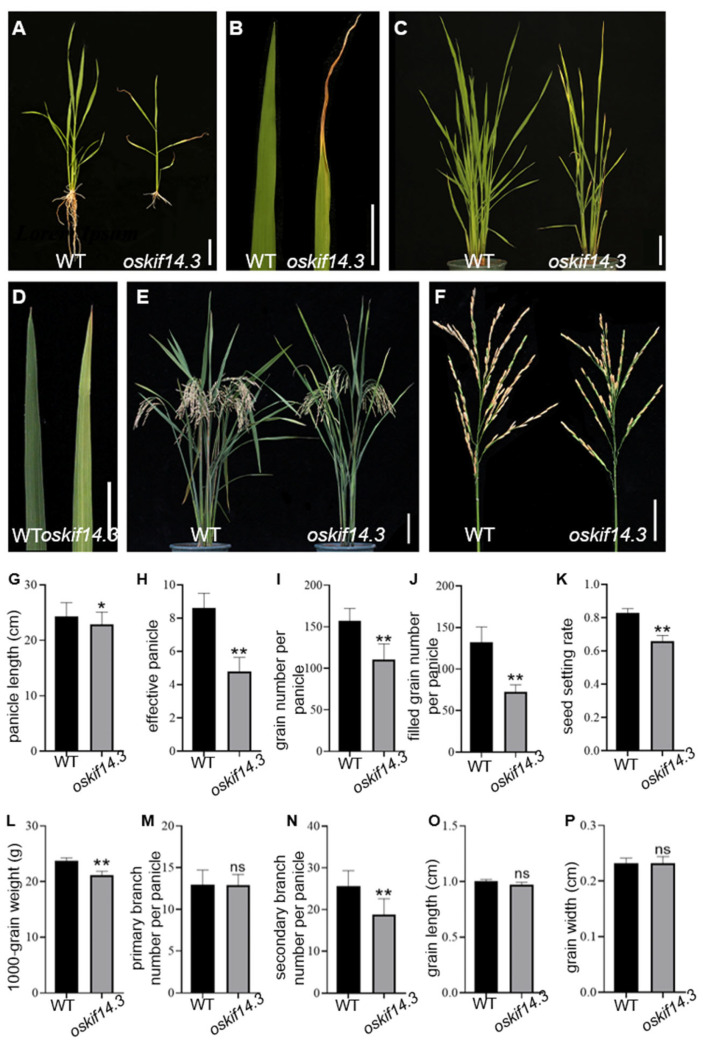
Phenotype analysis of the wild type (WT) and *oskif14.3* mutant. (**A**,**B**) phenotypes of wild type and *oskif14.3* plants and leaf tips at the seeding stage; bar = 5 cm (**A**), bar = 2.5 cm (**B**); (**C**,**D**) phenotypes of wild type and *oskif14.3* plants and leaf tips at booting stage; bar = 10 cm (**C**), bar = 2 cm (**D**); (**E**,**F**) phenotypes of wild type and *oskif14.3* plants and main panicle at maturity; bar = 10 cm (**E**), bar = 5 cm (**F**). (**G**) panicle length (cm); (**H**) effective panicle; (**I**) grain number per panicle; (**J**) filled grain number per panicle; (**K**) seed setting rate; (**L**) 1000-grain weight (g); (**M**) primary branch number per panicle; (**N**) second branch number per panicle; (**O**) grain length (cm); (**P**) grain width (cm); ns for non-significance results, * *p* < 0.05, ** *p* < 0.01 (Student’s *t*-test).

The *oskif14.3* mutant with stable genetic phenotype was screened out in the mutant library of EMS-induced maintainer line Xinong 1B. Compared with the wild type, it exhibited yellowish leaves from the seedling stage to the whole growth period (Figure 1A,B). The *oskif14.3* mutants also showed dwarfism and delayed heading days (Figure 1C–F). Various agronomic traits such as panicle length, effective panicles, grain number per panicle, filled grain number per panicle, seed setting rate, 1000-grain weight and secondary branch number per panicle were all decreased with significant difference in *oskif14.3* when compared with those of the wild type (Figure 1G–P). In *oskif14.3*, the chlorophyll a, chlorophyll b, total chlorophyll and carotenoid contents of the upper three leaves were all decreased with significant differences when compared with those of the wild type in the seedling stage (Table 1).

**Figure 2:**
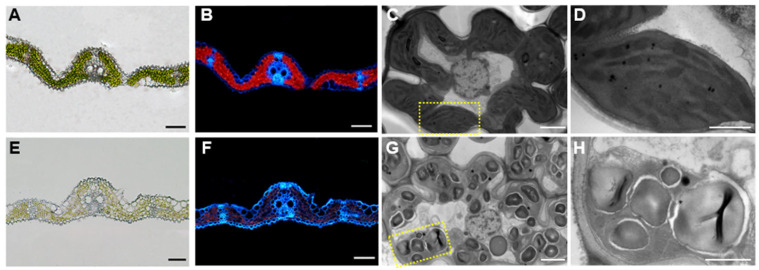
Photosynthetic pigment analysis of the wild type (WT) and *oskif14.3* mutant. (**A**,**B**) transverse sections of leaves in wild type under white light (**A**) and UV light at 488 nm (**B**); (**C**,**D**) ultrastructure of chloroplasts in wild type at the tillering stage, where the yellow box in (**C**) is enlarged as (**D**); (**E**,**F**) transverse sections of leaves in *oskif14.3* under white light (**E**) and UV light at 488 nm (**F**); (**G**,**H**) ultrastructure of chloroplasts in *oskif14.3* mutant at the tillering stage, where the yellow box in (**G**) is enlarged as (**H**); bars in (**A**,**B**,**E**,**F**) = 5 μm, bars in (**C**,**G**) = 2 μm, bars in (**D**,**H**) = 1 μm.

Cytological observations with freezing-section and fluorescence microscope revealed that the chlorophyll in *oskif14.3*’s leaves was nearly degraded, which led to a pale green leaf section and failed to emit red fluorescence under the 488 nm ultraviolet light (Figure 2A,B,E,F). Cytological observation with ultra-section and transmission electron microscopy was carried out and the results further demonstrated excessive starch granule accumulation, compressed and broken stroma lamellae in the chloroplast of *oskif14.3*’s leaves compared to the wild type (Figure 2C,D,G,H).

**Figure 3:**
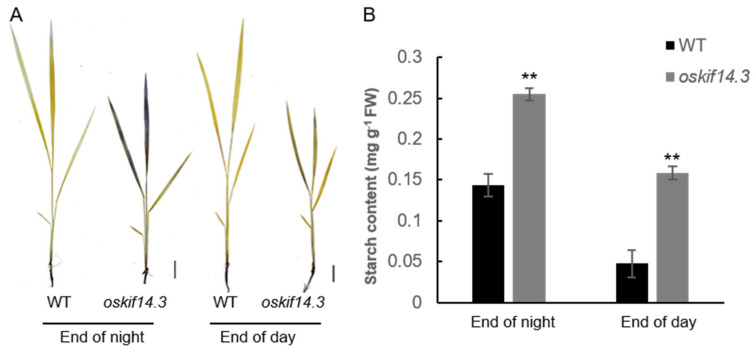
Excessive starch accumulation was observed in the *oskif14.3*’s leaves compared to the wild type (WT). (**A**) I_2_/KI staining of the wild type and oskif14.3 mutant during the fourth leaf stage; (**B**) starch content measurement of the wild type and oskif14.3 mutant during the fourth leaf stage. ** *p* < 0.01 (Student’s *t*-test).

For further analysis, I_2_/KI staining of starch was carried out. At both the end of the day and the end of the night, the *oskif14.3*’s leaves exhibited deeper staining than those of the wild type, indicating excessive starch accumulation in *oskif14.3*’s leaves (Figure 3A). Measurement of starch content indicated that more starch was accumulated in the *oskif14.3*’s leaves than those of the wild type (Figure 3B).

Triose phosphate/phosphate translocator OsTPT1 and OsTPT2 were located in the inner envelope membrane of plant chloroplast and catalyze the counter exchange of triose phosphate/3-phosphoglycerate for phosphate [24]. Large subunit of ADP-glucose pyrophosphorylase *OsAGPL1*, *OsAGPL2* and *OsAGPL3* function in starch biosynthesis [25,26]; α-glucan water dikinase *OsGWD1* and debranching enzyme isoamylase3 *OsISA3* function in starch degradation [27,28]. The expression of *OsTPT1*, *OsTPT2*, *OsGWD1* and *OsISA3* was significantly lower in *oskif14.3*’s leaves than those of the wild type, while the expressions of *OsAGPL1*, *OsAGPL2* and *OsAGPL3* were significantly increased in *oskif14.3*’s leaves (Figure S1). These results indicated that the starch metabolism was altered in *oskif14.3*.

### 2.2. Map-Based Clone of OsKIF14.3

**Figure 4:**
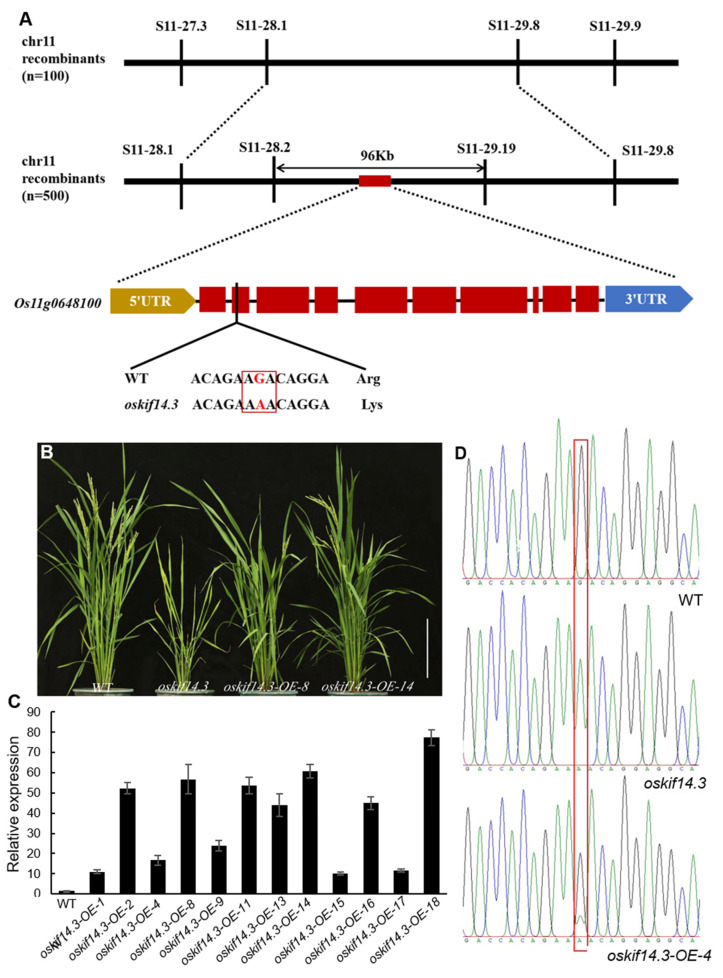
Map-based clone of *OsKIF14.3*. (**A**) Map-based clone of *OsKIF14.3*; (**B**) phenotypes of wild type (WT), *oskif14.3* mutant and overexpressed transgenic plants at the seeding stage; bar = 20 cm; (**C**) the expression level of *OsKIF14.3* in the overexpressed transgenic plants; (**D**) the sequencing results of wild type, *oskif14.3* mutant and overexpressed transgenic plants at mutation sites.

In our study, the heterozygous mutant *oskif14.3*/+ exhibited a slight yellowing leaf phenotype, suggesting that the *oskif14.3*’s traits were inherited in a semi-dominant manner (Figure S2). When the mutant was backcrossed with 1B, the F_2_ population resulting from self-pollination had a segregation ratio of 143:301:155 (normal plants: slight yellowing plants: yellowish plants), close to the expected 1:2:1 segregation ratio (χ^2^ = 0.503) for a semi-dominant single locus. Map-based cloning was employed to identify the gene responsible for the mutant phenotype (Figure 4A). The mutant was crossed to the restoring line Jinhui10. The normal plants were selected from the F_2_ population for mapping. The *OsKIF14.3* was finally located between SSR marker S11-28.2 and S11-29.19, with a physical distance of 96 kb (Figure 4A). Within this region, there were 11 annotated genes. These genes were all sequenced and a single nucleotide mutation from G to A leading to the 55 amino acid change from Arg to Lys in *Os11g0648100* occurring in the *oskif14.3* genome compared to the wild type (Figure 4A). To confirm whether the single nucleotide substitution of *Os11g0648100* was responsible for the defective phenotypes, we transformed the *oskif14.3* plants with the complete coding sequence of *Os11g0648100* under the control of ubiquitin promoter mediated by *Agrobacterium.* Twelve positive transgenic plants were obtained with polymerase chain reaction (PCR) and β-glucuronidase (GUS) activity detection. Moreover, *OsKIF14.3OE* plants with an overexpressed expression level of *Os11g0648100* and polymorphism (G/A) at the mutational site exhibited a normal phenotype similar to the wild type (Figure 4B–D). These results demonstrated that *Os11g0648100* was the functional gene of *OsKIF14.3*.

### 2.3. Characteristic Analysis of OsKIF14.3 Gene

**Figure 5:**
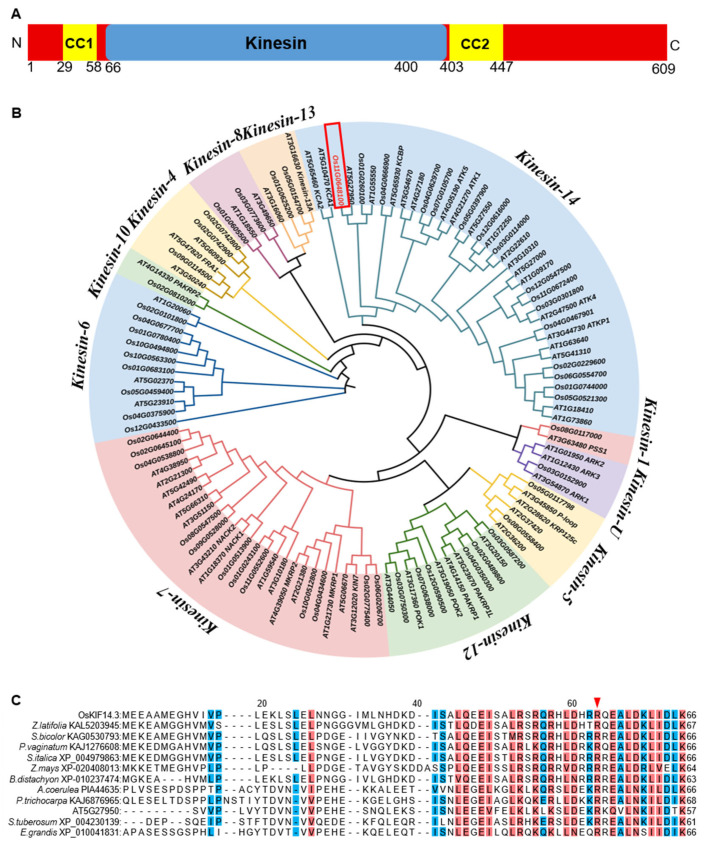
Bioinformatic analysis of OsKIF14.3. (**A**) Prediction of protein structure of OsKIF14.3; (**B**) phylogenetic tree analysis of Kinesin family between rice and Arabidopsis; (**C**) homologous analysis of CC1 domain among different species; the red triangle in (**C**) represent the mutation site in the *oskif14.3*.

Bioinformatic analysis revealed that *OsKIF14.3* encoded a kinesin protein with a conserved motor domain and two coiled-coil domains and belonged to the kinesin-14 family (Figure 5A,B). Furthermore, the mutation from Arg to Lys occurred on the first coiled coil in oskif14.3, which is also conserved among different species (Figure 5C).

**Figure 6:**
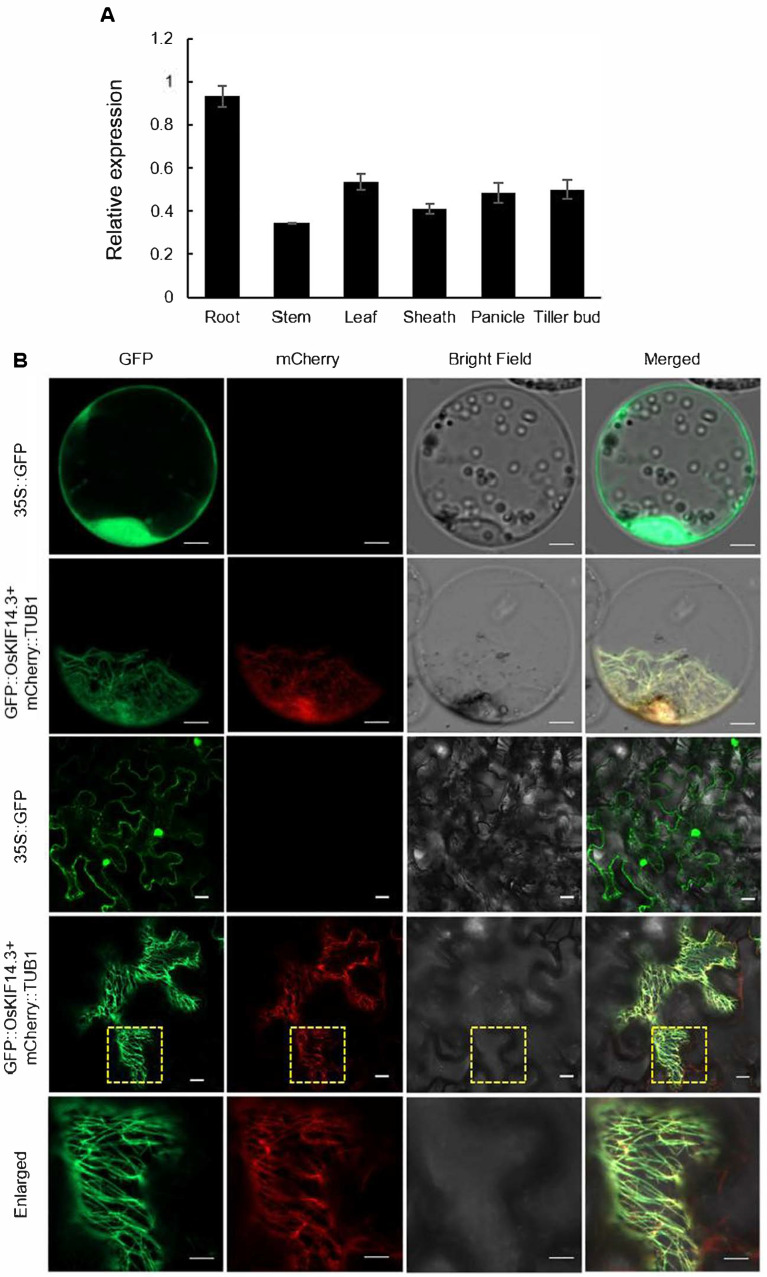
Gene characteristic analysis of OsKIF14.3. (**A**) Expression pattern of *OsKIF14.3*; (**B**) subcellular location of OsKIF14.3 and OsKIF14.3 colocalizes with microtubule marker TUB1; bar = 20 μm.

The expression pattern of *OsKIF14.3* in the wild type was analyzed with quantitative real-time PCR at the heading stage. The results revealed that *OsKIF14.3* was constitutively expressed in the roots, stems, young leaves, leaf sheathes, young panicles, and tiller buds in the wild type (Figure 6A). 

In order to determine the subcellular location of OsKIF14.3, a dual-expression vector pCAMBIA1300::*CamV35S*::*GFP*::*OsKIF14.3* was constructed and co-transformed with the microtubule marker pCAMBIA1300::*CamV35S*::*mCherry*::*OsKIF14.3* into both the rice protoplasts and tobacco leaf epidermal cells, respectively. Green fluorescence emitted by the GFP::OsKIF14.3 fusion protein overlapped with the red fluorescence emitted by the microtubule marker mCherry::TUB1 in both the protoplasts and tobacco leaves (Figure 6B). These results indicate that OsKIF14.3 is localized on the microtubule.

### 2.4. OsKIF14.3 Functions as Homodimer

**Figure 7:**
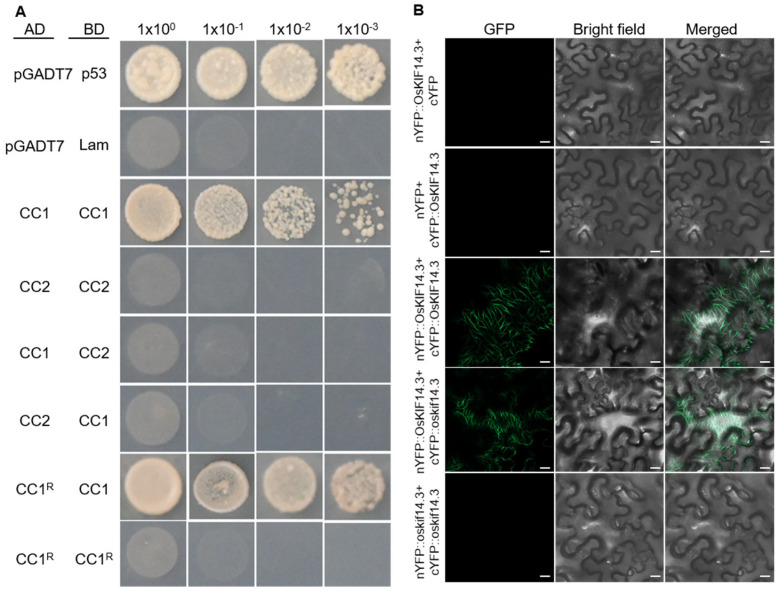
OsKIF14.3 functions as homodimer. (**A**) Verification of the interaction between CC1 (29–58 aa) and CC2 (403–447 aa) by yeast two-hybrid assay. CC1^R^ represents the CC1 (29–58 aa) in the *oskif14.3* mutant. (**B**) The bimolecular fluorescence complementation assay verified that OsKIF14.3 can form homodimers in vivo; bar = 20 μm.

The Kinesin superfamily always functions as homodimer or heterodimer via the two coiled coils [29]. On OsKIF14.3, one coiled coil (CC1: 29–58 aa) is located on the N terminal of the motor domain and the other coiled coil (CC2: 403–447 aa) localized on the C terminal of the motor domain (Figure 5A). CC1 and CC2 were amplified and cloned into pGBKT7 and pGADT7, respectively, and the yeast two-hybrid system was carried out. CC1 could interact with CC1, while CC2 could not interact with CC2 (Figure 7A). Moreover, CC1 could interact with the mutation form CC1^R^, but CC1^R^ could not interact with CC1^R^ itself (Figure 7A). To further analyze, BiFC assay was carried out. nYFP-OsKIF14.3 could interact with cYFP-OsKIF14.3 and cYFP-oskif14.3, respectively, to emit fluorescence signal on the microtubules in tobacco, while no fluorescence signal was observed when nYFP-oskif14.3 was cotransformed with cYFP-oskif14.3 (Figure 7B). These results indicate that OsKIF14.3 functions as a homodimer in vivo and in vitro, and the conserved R55 on the CC1 domain is critical for OsKIF14.3’s dimerization.

### 2.5. OsKIF14.3 Participates in the Plasma Membrane Location of OsSWEET11

The *oskif14.3* mutant accumulates excessive starch in the yellowish leaves and one of kinesin’s main functions is transporting secretary protein [9,10]. To explore the regulation mechanism of *OsKIF14.3*, subcellular location of sugar transporters OsSUT1, OsSUT2, OsSWEET5, OsSWEET11 and OsSWEET14 was analyzed in the protoplast of the wild type and *oskif14.3* mutant. The results revealed that OsSUT1, OsSUT2, OsSWEET5, OsSWEET14 showed no change in the subcellular location between the wild type and the *oskif14.3* mutant (Figure S3). OsSUT1 and OsSWEET14 were located on the plasma membrane in both the wild type and the *oskif14.3* mutant (Figure S3). OsSUT2 and OsSWEET5 were located on the vacuole in both the wild type and the *oskif14.3* mutant (Figure S3). OsSWEET11 was located on the plasma membrane in the wild type, which is consistent with a previous report [30], but it was located both in the cytoplasm and on the plasma membrane in *oskif14.3* (Figure S3). These results indicate that OsKIF14.3 functions in the starch metabolism via regulating the subcellular location of OsSWEET11.

## 3. Discussion

### 3.1. The oskif14.3 Is a New Leaf Color Mutant

Rice leaf is the most important organ and the color of rice leaves is positively correlated with photosynthetic efficiency, which determines the production and accumulation of organic substance. Leaf color mutants including albino mutants, white stripe mutants, zebra mutants, pale green mutants, yellowish mutants, etc., and these were ideal materials to study the development process and function of leaves [6]. Nowadays, a large number of leaf color genes have been cloned and thoroughly depicted and most of these genes functioned in chlorophyll metabolism, such as *YGL1*, *OsCHLH*, *OsCHLD*, *OsCAO1*, *OsCAO2*, *OsPDS*, *OsZDS*, etc., while some of these genes functioned in chloroplast assembly, such as *V1*, *V2*, *NTRC*, *YGL8*, etc. Furthermore, *V3*, *ST1*, *OsDOS*, and *OsSAC3* participated in leaf color regulation independent of chlorophyll and chloroplast [1,2,5,7,31,32,33,34,35,36,37,38]. *OsSAC3* encodes the xanthine dehydrogenease, which functions in uric acid metabolism. Mutation of *OsSAC3* caused the decreased expression of *OsSWEET3a*, *OsSWEET6a* and *OsSWEET14*, and altered carbohydrate distribution, resulting in sucrose and starch accumulation in the *ossac3* mutant [7]. The *oskif14.3* mutant showed similar starch accumulation to the *ossac3* mutant. *OsKIF14.3*, which encodes a kinesin on the microtubule, plays a role in starch metabolism via controlling the subcellular location of OsSWEET11. Thus, OsKIF14.3 might participate in leaf color regulation independent of the chlorophyll- and chloroplast-related pathway. Similar to *ossac3*, excessive starch granule accumulated in *oskif14.3*’s chloroplast, leading to broken thylakoid stroma lamella and decreased photosynthetic pigment, and resulting in yellowish leaf phenotype [7].

### 3.2. OsKIF14.3 Participates in Carbohydrate Distribution by Regulating OsSWEET11’s Location

As the common molecular motor, the members of kinesin superfamily convert chemical energy to mechanical energy and function in cell cycle, cell wall deposition, organelle movement, secretary protein transportation, etc. [8,9,10]. OsKIF14.3 belonged to the Kinesin-14 subfamily, which was independent of starch metabolism. In *oskif14.3*’s leaves, excessive starch accumulation was observed and genes in starch metabolism showed altered expression (Figure 7A). Thus, *OsKIF14.3* might function in starch metabolism by regulating sugar transporter location. OsSUT1 localized on the plasma membrane, showed sucrose transporter activity and functioned in sucrose loading in the phloem. The *ossut1* mutant showed reduced growth and grain yield [39]. OsSUT2 functions in sucrose uptake from the vacuole and localized to the tonoplast. The *ossut2* mutant exhibited a growth retardation phenotype [40]. OsSWEET5 encoded a galactose transporter which localized on the plasma membrane. OsSWEET5-overeexpressing plants displayed the phenotypes of growth retardation and previous senescence at the seedling stage [41]. Both OsSWEET11 and OsSWEET14 encoded sucrose transporter on the plasma membrane and work together to mediate sucrose loading in the phloem of rice leaves [42,43]. The subcellular location of OsSUT1, OsSUT2, OsSWEET5 and OsSWEET14 showed no change between the wild type and *oskif14.3*, while OsSWEET11 was located on the plasma membrane in the wild type and was located both on the plasma membrane and in the cytoplasm in the *oskif14.3*. The location of OsSWEET11 was altered in the *oskif14.3*. Thus, the kinesin-14 family member, OsKIF14.3, participates in starch metabolism by regulating the subcellular location of OsSWEET11.

## 4. Materials and Methods

### 4.1. Plant Materials and Growth Conditions

The Xinong 1B, *oskif14.3* mutant and Jinhui10 used in this study were all indica rice belonging to our laboratory. In this study, all experimental materials were planted at the experimental base of the Rice Research Institute, Southwest University, in Xiema Town, Beibei District, Chongqing, China. The materials were sown in mid-March and transplanted in mid-April. A block design with a 10 × 10 configuration was used for field planting, with plant spacing set at 16.67 cm and 20.00 cm. At maturity, ten non-boundary plants each were selected from WT and *oskif14.3* to reduce edge effects, for the investigation of agronomic traits. Experimental data were statistically analyzed.

### 4.2. Physical and Chemical Analysis

To measure the pigment content, 0.1 g of mixed leaf tips from the 1st–3rd inverted leaves were collected. The photosynthetic pigment content was quenched and measured following the method of [7]. During the fourth leaf stage, 0.1 g leaf tips were collected and starch content measurement was conducted following the manufacturer’s protocol (E2ST-100, Bioassy system, Bay Area, CA, USA).

### 4.3. Frozen Section

During the tillering stage, leaf samples from the same part of WT and *oskif14.3* plants were collected. Samples were quickly frozen and embedded in liquid nitrogen with a freezing embedding agent. Then, the embedded materials were cut into 8 μm thick slices at −10 °C using the freezing microtome CRYOSTAR NX50 (Thermo Scientific, Waltham, MA, USA). The slices were picked up with a glass slide. After washing away the embedding agent with ddH_2_O, chloroplast morphology and autofluorescence were observed under white and ultraviolet light using a DM6B Digital slide scanning system (Leica, Wetzal, Germany).

### 4.4. Transmission Electron Microscope (TEM)

During the tillering stage, 1 mm × 3 mm leaf samples were excised from WT and *oskif14.3* using a blade. Samples were fixed overnight at 4 °C in 3.5% glutaraldehyde, then rinsed three times with 0.1 M PBS for 3 min each. Samples underwent ethanol dehydration in gradients of 30%, 50%, 60%, 70%, 80%, 90%, 95%, and 100%, 15 min per step. After dehydration, they were replaced six times with 100% acetone, 30 min per replacement. Infiltration was performed successively at 3:1, 1:1, and 1:3 (acetone: embedding agent) gradients for 12 h each. Infiltrated samples were embedded, and the blocks polymerized at 65 °C for one week. Ultrathin sections were prepared from the blocks, double-stained with uranyl acetate and lead citrate, and observed under a transmission electron microscope.

### 4.5. Map-Based Cloning

F_1_ plants were obtained by crossing *oskif14.3* with indica restore line Jinhui10. The F_1_ plants self-crossed to yield F_2_ plants, and a chi-square test analyzed the F_2_ segregation ratio. Recessive homozygous F_2_ plants and polymorphic molecular markers across 12 rice chromosomes screened target gene-linked markers via polyacrylamide gel electrophoresis. Additional markers were designed around the linked ones while expanding the F_2_ population, narrowing the target gene interval according to the decline in heterozygous plants. Sequencing and analysis of the gene sequences within the mapped interval identified candidate genes.

### 4.6. Statistical Analysis

The quantity of experimental samples and replicates followed the specifications in the article’s figure legends. Data are presented as means ± SD. Student’s *t*-test was applied for statistical analysis. Significance is indicated by asterisks: * for *p* < 0.05, ** for *p* < 0.01, and ns for non-significant results.

### 4.7. Multiple Sequence Alignment and Evolutionary Analysis

Protein sequences applied in multiple sequence alignment and phylogenetic tree construction were acquired by searching the Phytozome (https://phytozome-next.jgi.doe.gov/; accessed on 1 December 2024) and NCBI (https://www.ncbi.nlm.nih.gov/; accessed on 1 December 2024) using the OsKIF14.3 amino acid sequence as a query. Multiple sequence alignment was performed with Clustal X (1.83) software using multiple alignment modes. Evolutionary analysis was conducted in MEGA X (11.0.11).

### 4.8. Function Analysis of Os11g0648100

To explore the function of *OsKIF14.3*, the complete coding sequence of *Os11g0648100* was amplified and cloned into binary pCAMBIA1301 under the control of the ubiquitin promoter. The accuracies of the constructs were confirmed by sequencing, and the constructs were transformed into the *oskif14.3* callus mediated by *A. tumefaciens* EHA105. Transformants were screened out under hygromycin and verified by GUS activity detection and sequencing. The sequenced coding sequence of *Os11g0648100* is presented in Figure S4.

### 4.9. Gene Expression and RNA Isolation

Total RNA was isolated from the roots, stems, leaves, sheathes, panicles and tiller buds of the wild type using the RNAprep Pure Plant RNA Purificatiion Kit (Tiangen, Beijing, China). The first-strand cDNA was synthesized with 1 µg of total RNA using oligo (dT)18 as primers in a 20 µL reaction volume using the SuperScript III Reverse Transcriptase Kit (Invitrogen, Carlsbad, CA, USA). Quantitative real-time PCR analysis was performed with a Bio-Rad Real-Time PCR System (Bio-Rad, Hercules, CA, USA), the SYBR Premix Ex Taq II Kit (TaKaRa, Tokyo, Japan) and gene-specific primers (Table S1). Ubiquitin (*Ubq5*) was used as an endogenous control. At least three replicates were performed.

### 4.10. Subcelluar Localization of GFP::OsKIF14.3 in Protoplast and N. benthamiana Cells

To conduct the subcellular location analysis of OsKIF14.3, the WT OsKIF14.3 CDS fragments were cloned into the pCAMBIA1300-GFP under the control of the *CAMV35S* promoter to generate a fusion construct with GFP at the N terminus. GFP::OsKIF14.3 and microtubule marker were cotransformed into rice protoplast mediated by PEG and co-infiltrated into leaves of *N*. *benthamiana*. Fluorescence signals were sectioned and examined using a confocal laser scanning microscope (LSM510 META, Zeiss, Jena, Germany).

### 4.11. Bimolecular Fluorescence Complementation Assay

The full-length cDNA of *OsKIF14.3* and oskif14.3 was subcloned into the both Pxy-nYFP and pXY-cYFP. For expression assays, *A. tumefaciens* (strain GV3101) carrying the BiFC constructs was used for infiltration of 6- to 7-week-old *N. benthamiana* leaves. The YFP fluorescence was visualized with a confocal scanning microscope after infiltration for 48–72 h.

### 4.12. Protein Interaction Analysis

Yeast two-hybrid assays were performed using the Matchmaker Gold Yeast Two Hybrid System (Clontech, Mountain View, CA, USA). The CC1, CC1^R^ and CC2 were amplified and ligated into the yeast expression vector pGBKT7, and pGADT7 (Clontech). The yeast strain used in the assays was Y2HGold. The vector pGADT7-T plus pGBKT7-lam served as a negative control and pGADT7-T plus pGBKT7-p53 as a positive control. These plasmids were co-transformed into Y2HGold in an AD-BD-coupled manner. Detailed procedures are described in the manufacturer’s instructions (Yeast Protocols Handbook, PT3024-1; Clontech).

## 5. Conclusions

This study has functional analysis of a semi-dominant mutant, *oskif14.3*, which exhibited a yellowish leaf phenotype with increased starch accumulation. The *OsKIF14.3* gene was map-based cloned and functional verified. The *OsKIF14.3* encodes a typical kinesin protein which targets on the microtubule and functions as homodimers. Mutation of OsKIF14.3 causes a defect in its dimerization, leading to the misdistribution of OsSWEET11 (OsSWEET11 was localized on the plasma membrane in the wild type, but both on the plasma membrane and cytoplasm in the *oskif14.3* mutant), resulting in increased starch accumulation and yellowish leaves in the *oskif14.3* mutant. These results enriched our understanding of the kinesin superfamily and leaf color regulation mechanism.

## Figures and Tables

**Table 1:** Photosynthetic pigment contents of the leaves of wild type (WT) and *oskif14.3* at seedling stage.

Leaf Position	Materials	Chla	Chlb	Total chl	Car
The first leaf from the top	WT	5.27 ± 0.16	2.36 ± 0.51	7.26 ± 0.60	2.18 ± 0.09
	*oskif14.3*	3.32 ± 0.19 **	0.73 ± 0.03 *	4.05 ± 0.23 **	1.30 ± 0.07 **
The second leaf from the top	WT	5.25 ± 0.05	2.41 ± 0.10	4.05 ± 0.14	2.18 ± 0.04
	*oskif14.3*	3.82 ± 0.35 *	0.79 ± 0.02 *	4.68 ± 0.48 **	1.46 ± 0.18 *
The third leaf from the top	WT	5.90 ± 0.44	1.56 ± 0.26	7.45 ± 0.57	2.12 ± 0.33
	*oskif14.3*	4.61 ± 0.20 *	0.96 ± 0.04 *	5.56 ± 0.22 *	1.49 ± 0.06 *

* *p* < 0.05, ** *p* < 0.01 (Student’s *t*-test).

## Data Availability

The data presented in this study are available on request from the corresponding author. The provided materials are for scientific research purposes only.

## Supplementary Materials

The following supporting information can be downloaded at: https://www.mdpi.com/article/10.3390/ijms262311577/s1.

## References

[1] Lee S., Kim J., Yoo E., Lee C., Hirochika H., An G. (2005). Differential regulation of *chlorophyll a oxygenase* genes in rice. Plant Mol. Biol..

[2] Ruan B., Gao Z., Zhao J., Zhang B., Zhang A., Hong K., Yang S., Jiang H., Liu C., Chen G. (2017). The Rice *YGL* Gene Encoding an Mg^2+^-chelatase ChlD Subunit is Affected by Temperature for Chlorophyll Biosynthesis. J. Plant Biol..

[3] Sakuraba Y., Rahman M., Cho S., Kim Y., Koh H., Yoo S., Paek N. (2013). The rice faded green leaf locus encodes protochlorophyllide oxidoreductaseB and is essential for chlorophyll synthesis under high light conditions. Plant J..

[4] Wang Q., Chen P., Wang H., Chao S., Guo W., Zhang Y., Miao C., Yuan H., Peng B. (2023). Physiological and transcriptomic analysis of OsLHCB3 knockdown lines in rice. Mol. Breed..

[5] Yoo S., Cho S., Sugimoto H., Li J., Kusumi K., Koh H., Iba K., Paek N. (2009). Rice *Virescent3* and *Stripe1* Encoding the Large and Small Subunits of Ribonucleotide Reductase Are Required for Chloroplast Biogenesis during Early Leaf Development. Plant Physiol..

[6] Li W., Zhang Y., Mazumder M.A.R., Pan R., Akhter D. (2022). Research progresses on rice leaf color mutants. Crop Des..

[7] Xie Z., Zhao B., Zhang M., Sang X., Zhao F., Feng P., He G., Zhu X. (2022). Mutation of *OsSAC3*, Encoding the Xanthine Dehydrogenase, Caused Early Senescence in Rice. Int. J. Mol. Sci..

[8] Endow S., Kull F., Liu H. (2010). Kinesins at a glance. J. Cell Sci..

[9] Vale R., Reese T., Sheetz M. (1985). Identification of a Novel Force-Generating Protein, Kinesin, Involved in Microtubule-Based Motility. Cell.

[10] Li J., Xu Y., Chong K. (2012). The novel functions of kinesin motor proteins in plants. Protoplasma.

[11] Vale R., Fletterick R. (1997). The design plan of kinesin motors. Annu. Rev. Cell Dev. Biol..

[12] Lawrence C., Malmberg R., Muszynski M., Dawe R. (2002). Maximum likelihood methods reveal conservation of function among closely related kinesin families. J. Mol. Evol..

[13] Chen C., Marcus A., Li W., Hu Y., Calzada J., Grossniklaus U., Cyr R., Ma H. (2002). The Arabidopsis ATK1 gene is required for spindle morphogenesis in male meiosis. Development.

[14] Marcus A., Li W., Ma H., Cyr R. (2003). A kinesin mutant with an atypical bipolar spindle undergoes normal mitosis. Mol. Biol. Cell.

[15] Tian J., Han L., Feng Z., Wang G., Liu W., Ma Y., Yu Y., Kong Z. (2015). Orchestration of microtubules and the actin cytoskeleton in trichome cell shape determination by a plant-unique kinesin. eLife.

[16] Ganguly A., Zhu C., Chen W., Dixit R. (2020). FRA1 Kinesin Modulates the Lateral Stability of Cortical Microtubules through Cellulose Synthase-Microtubule Uncoupling Proteins. Plant Cell.

[17] Suetsugu N., Sato Y., Tsuboi H., Kasahara M., Imaizumi T., Kagawa T., Hiwatashi Y., Hasebe M., Wada M. (2012). The KAC Family of Kinesin-Like Proteins is Essential for the Association of Chloroplasts with the Plasma Membrane in Land Plants. Plant Cell Physiol..

[18] Zhou S., Wang Y., Li W., Zhao Z., Ren Y., Wang Y., Gu S., Lin Q., Wang D., Jiang L. (2011). *Pollen Semi-Sterility1* Encodes a Kinesin-1-Like Protein Important for Male Meiosis, Anther Dehiscence, and Fertility in Rice. Plant Cell.

[19] Zhou Y., Li Y., You H., Chen J., Wang B., Wen M., Zhang Y., Tang D., Shen Y., Yu H. (2024). Kinesin-1-like protein PSS1 is essential for full-length homologous pairing and synapsis in rice meiosis. Plant J..

[20] Zhang M., Zhang B., Qian Q., Yu Y., Li R., Zhang J., Liu X., Zeng D., Li J., Zhou Y. (2010). Brittle Culm 12, a dual-targeting kinesin-4 protein, controls cell-cycle progression and wall properties in rice. Plant J..

[21] Deng Z., Liu L., Li T., Yan S., Kuang B., Huang S., Yan C., Wang T. (2015). OsKinesin-13A Is an Active Microtubule Depolymerase Involved in Glume Length Regulation via Affecting Cell Elongation. Sci. Rep..

[22] Fang J., Yuan S., Li C., Jiang D., Li X. (2018). Reduction of ATPase activity in the rice kinesin protein Stemless Dwarf1 inhibits cell division and organ development. Plant J..

[23] Tseng K., Wang P., Lee Y., Bowen J., Gicking A., Guo L., Liu B., Qiu W. (2018). The preprophase band-associated kinesin-14 OsKCH2 is a processive minus-end-directed microtubule motor. Nat. Commun..

[24] Wang Q., Chen J., Wang X., Sun J., Sha W. (2002). Molecular cloning and expression analysis of the rice triose phosphate/phosphate translocator gene. Plant Sci..

[25] Akihiro T., Mizuno K., Fujimura T. (2005). Gene expression of ADP-glucose pyrophosphorylase and starch contents in rice cultured cells are cooperatively regulated by sucrose and ABA. Plant Cell Physiol..

[26] Lee S., Hwang S., Han M., Eom J., Kang H., Han Y., Choi S., Cho M., Bhoo S., An G. (2007). Identification of the ADP-glucose pyrophosphorylase isoforms essential for starch synthesis in the leaf and seed endosperm of rice (*Oryza sativa* L.). Plant Mol. Biol..

[27] Hirose T., Aoki N., Harada Y., Okamura M., Hashida Y., Ohsugi R., Miyao A., Hirochika H., Terao T. (2013). Disruption of a rice gene for α-glucan water dikinase, *OsGWD1*, leads to hyperaccumulation of starch in leaves but exhibits limited effects on growth. Front. Plant Sci..

[28] Yun M., Umemoto T., Kawagoe Y. (2011). Rice Debranching Enzyme Isoamylase3 Facilitates Starch Metabolism and Affects Plastid Morphogenesis. Plant Cell Physiol..

[29] Allingham J., Sproul L., Rayment I., Gilbert S. (2007). Vik1 modulates microtubule-Kar3 interactions through a motor domain that lacks an active site. Cell.

[30] Chu Z., Fu B., Yang H., Xu C., Li Z., Sanchez A., Park Y., Bennetzen J., Zhang Q., Wang S. (2006). Targeting xa13, a recessive gene for bacterial blight resistance in rice. Theor. Appl. Genet..

[31] Sugimoto H., Kusumi K., Tozawa Y., Yazaki J., Kishimoto N., Kikuchi S., Iba K. (2004). The *virescent*-2 mutation inhibits translation of plastid transcripts for the plastid genetic system at an early stage of chloroplast differentiation. Plant Cell Physiol..

[32] Kong Z., Li M., Yang W., Xu W., Xue Y. (2006). A novel nuclear-localized CCCH-type zinc finger protein, OsDOS, is involved in delaying leaf senescence in rice. Plant Physiol..

[33] Pérez-Ruiz J., Spínola M., Kirchsteiger K., Moreno J., Sahrawy M., Cejudo F. (2006). Rice NTRC is a high-efficiency redox system for chloroplast protection against oxidative damage. Plant Cell.

[34] Wu Z., Zhang X., He B., Diao L., Sheng S., Wang J., Guo X., Su N., Wang L., Jiang L. (2007). A chlorophyll-deficient rice mutant with impaired chlorophyllide esterification in chlorophyll biosynthesis. Plant Physiol..

[35] Fang J., Chai C., Qian Q., Li C., Tang J., Sun L., Huang Z., Guo X., Sun C., Liu M. (2008). Mutations of genes in synthesis of the carotenoid precursors of ABA lead to pre-harvest sprouting and photo-oxidation in rice. Plant J..

[36] Kusumi K., Sakata C., Nakamura T., Kawasaki S., Yoshimura A., Iba K. (2011). A plastid protein NUS1 is essential for build-up of the genetic system for early chloroplast development under cold stress conditions. Plant J..

[37] Zhou S., Sawicki A., Willows R., Luo M. (2012). C-terminal residues of *Oryza sativa* GUN4 are required for the activation of the ChlH subunit of magnesium chelatase in chlorophyll synthesis. FEBS Lett..

[38] Kong W., Yu X., Chen H., Liu L., Xiao Y., Wang Y., Wang C., Lin Y., Yu Y., Wang C. (2016). The catalytic subunit of magnesium-protoporphyrin IX monomethyl ester cyclase forms a chloroplast complex to regulate chlorophyll biosynthesis in rice. Plant Mol. Biol..

[39] Wang G., Wu Y., Ma L., Lin Y., Hu Y., Li M., Li W., Ding Y., Chen L. (2021). Phloem loading in rice leaves depends strongly on the apoplastic pathway. J. Exp. Bot..

[40] Eom J., Cho J., Reinders A., Lee S., Yoo Y., Tuan P., Choi S., Bang G., Park Y., Cho M. (2011). Impaired Function of the Tonoplast-Localized Sucrose Transporter in Rice, OsSUT2, Limits the Transport of Vacuolar Reserve Sucrose and Affects Plant Growth. Plant Physiol..

[41] Zhou Y., Liu L., Huang W., Yuan M., Zhou F., Li X., Lin Y. (2014). Overexpression of *OsSWEET5* in Rice Causes Growth Retardation and Precocious Senescence. PLoS ONE.

[42] Chen L., Qu X., Hou B., Sosso D., Osorio S., Fernie A., Frommer W. (2012). Sucrose Efflux Mediated by SWEET Proteins as a Key Step for Phloem Transport. Science.

[43] Chu Z., Yuan M., Yao L., Ge X., Yuan B., Xu C., Li X., Fu B., Li Z., Bennetzen J. (2006). Promoter mutations of an essential gene for pollen development result in disease resistance in rice. Genes Dev..

