# Supplementary material for: Mutation of OsKIF14.3, a Kinesin-14 Subfamily Protein, Altered Starch Metabolism and Caused Yellowish Leaf in Rice
Source: Int J Mol Sci. 2025 Nov 29;26(23):11577. doi: 10.3390/ijms262311577 (PMC12692232; doi:10.3390/ijms262311577)
Supplement: Supplementary file 1 [file ijms-26-11577-s001.zip › ijms-3954506-supplementary.pdf]

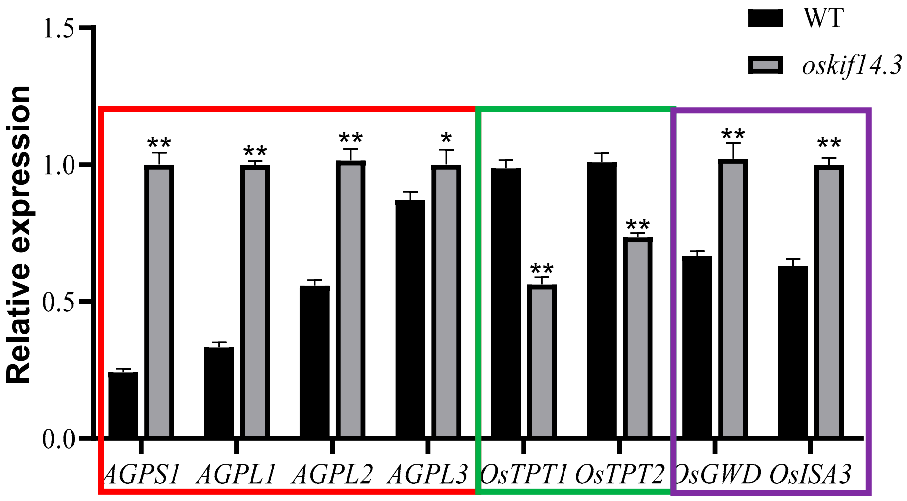

Supplementary Materials Figure S1. Quantitative real-time PCR analysis of starch metabolism-related genes of wild type and oskif14.3. *$p<0.05$, **$p<0.01$ (Student's *t*-test).

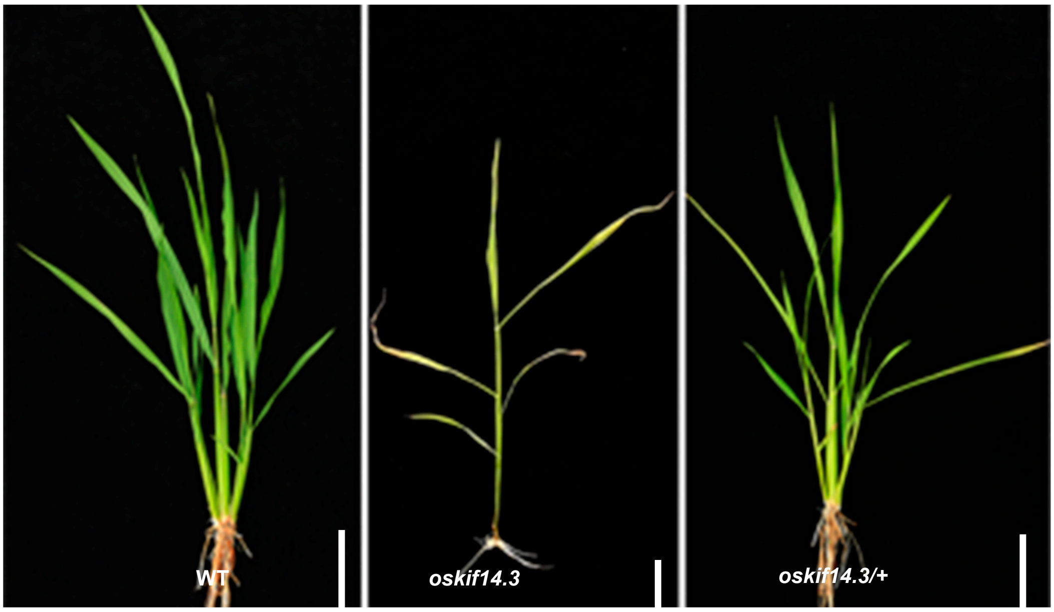

Supplementary Materials Figure S2. Plant phenotype of the wild type (WT), the oskif14.3 mutant and the oskif14.3/+ heterozygous plants. Bar=2cm.

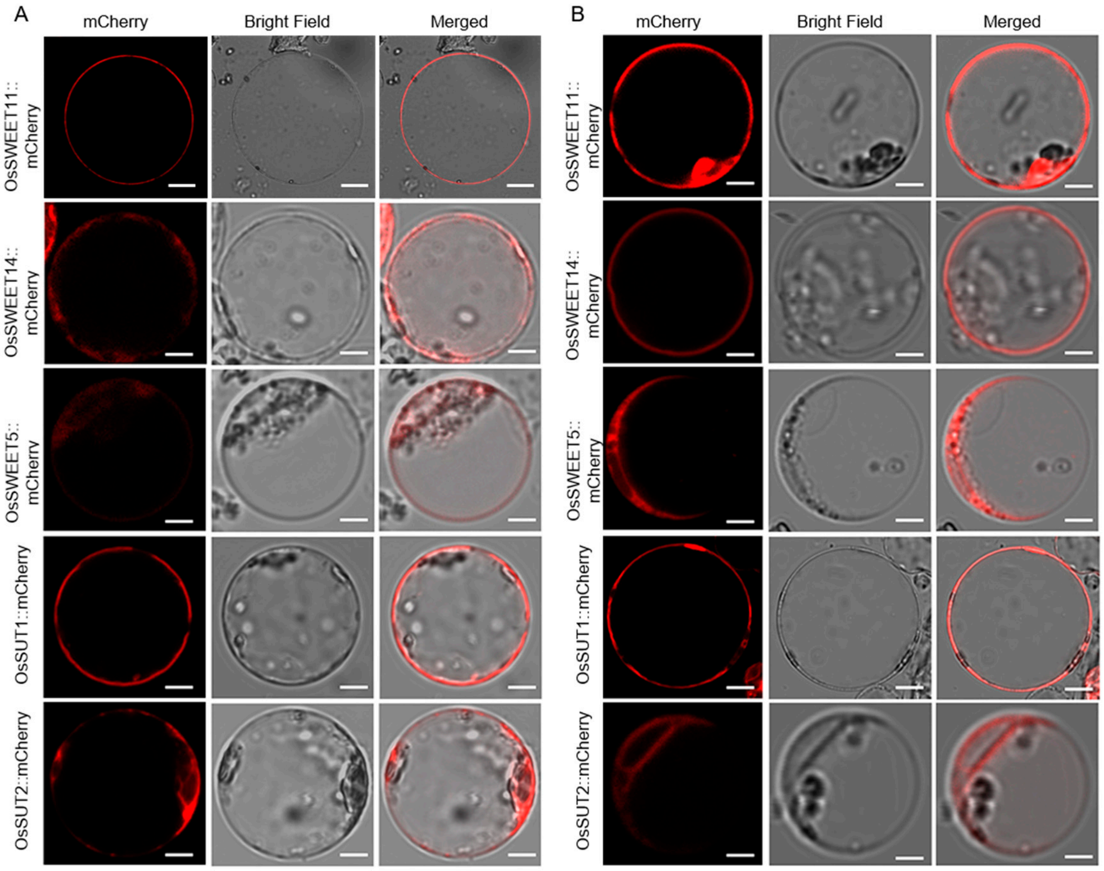

Supplementary Materials Figure S3. OsKIF14.3 participate in sugar metabolism by regulating the subcellular location of OsSWEET11. A: The subcellular localization of sugar transporter in wild type rice protoplasts; bar=10 μm; B: The subcellular localization of sugar transporter in *oskif14.3* rice protoplasts; bar=10 μm.

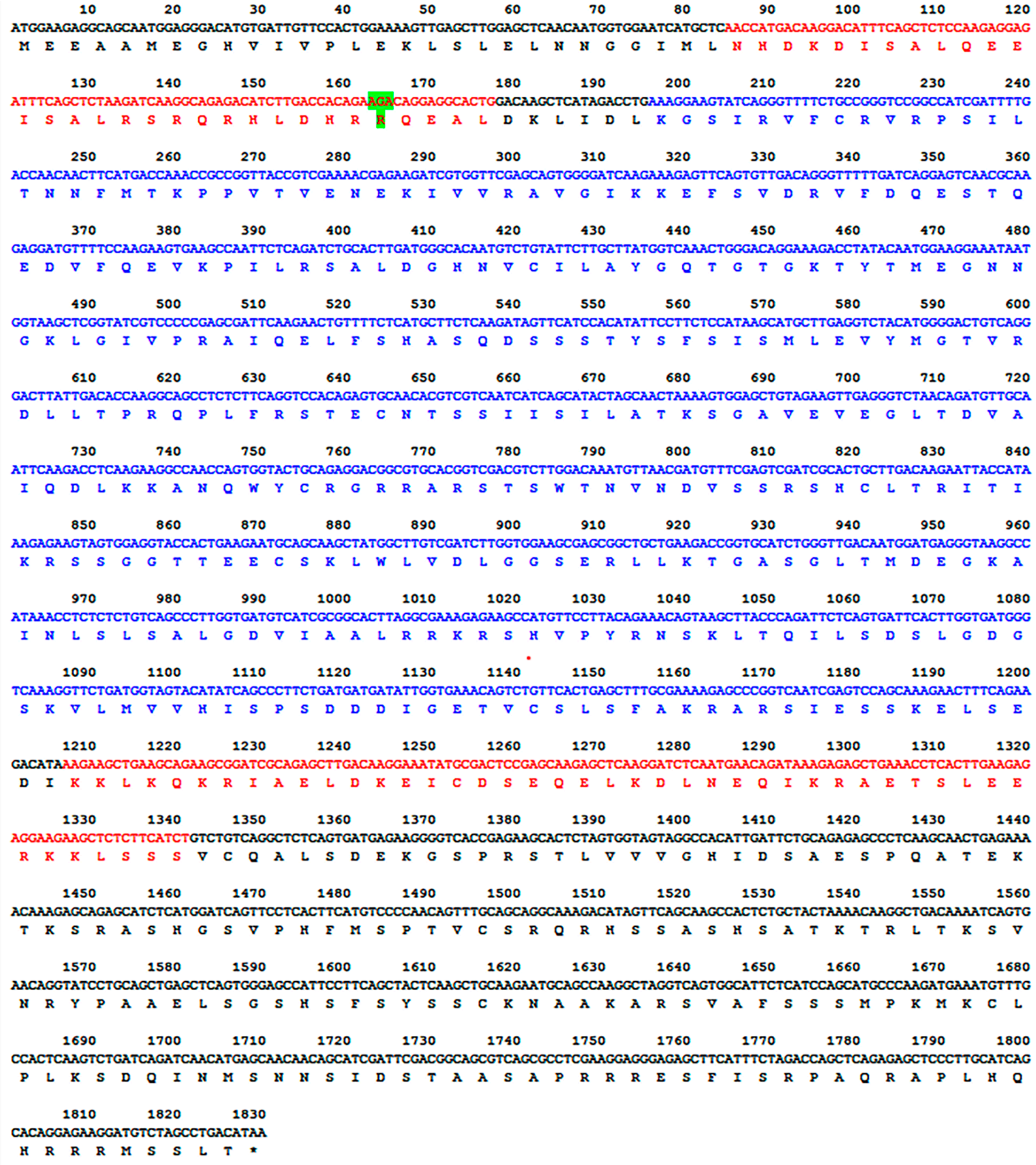

Supplementary Materials Figure S4. Coding sequence and predicted amino acids of OsKIF14.3 in Xinong1B. The 1830bp open reading frame of OsKIF14.3 in Xinong1B encodes a protein of 609 amino acids which contains a conserved motor domain and two coiled coil domains. The red-marked sequences are the gene and protein sequences of the coiled coil domain, the blue-marked sequences are the gene and protein sequences of the motor domain, and the green-background marked ones are the mutation sites; * for the termination codon.

Supplementary Materials Table S1 Primers used in this study

Function	Primer	Sequence (5'to 3')
Map-based clone of OsKIF14.3	S11-27.3-F	ACGGTGATGATTGCCACCG
	S11-27.3-R	AAATTCAAAATTATTCAAATTCAGTCA
	S11-28.1-F	CGTCTACCTGGTCTAATAGGATAATC
	S11-28.1-R	AGCCGTGCTTGACTCATTACT
	S11-28.2-F	GTGTGTGTTTATTCATCAAGTTCC
	S11-28.2-R	CACTTTTCCCAACTCGATACAC
	S11-29.19-F	TCAGCAGAGTGGGCTTCAAG
	S11-29.19-R	GATCAAGAAACACATACCCTACC
	S11-29.8-F	TCGTCTTACCGCAACAATCA
	S11-29.8-R	CTAACTCAACCACAACCATTTTAC
	S11-29.9-F	AGAGGGCCTTCTTGCCTTTAG
	S11-29.9-R	ATTATTTGATTGATGTAGGATTTGAG
Functional verification of OsKIF14.3	OsKIF14.1-OEC-F	GTGTTACTTCTGCAGGATCCATGGAAGAGGCAGCAATGGAGGG
	OsKIF14.1-OEC-R	CCCTTGCTCACCATGGATCCTGTCAGGCTAGACATCCTTCTCCTGTG
RT-qPCR	AGPS1-F	GTGCCACTTAAAGGCACCATT
	AGPS1-R	CCCACATTTCAGACACGGTTT
	AGPL1-F	GGAAGACGGATGATCGAGAAAG
	AGPL1-R	CACATGAGATGCACCAACGA
	AGPL2-F	AGTTCGATTCAAGACGGATAGC
	AGPL2-R	CGACTTCCACAGGCAGCTTATT
	AGPL3-F	AAGCCAGCCATGACCATTTG
	AGPL3-R	CACACGGTAGATTCACGAGACAA
	OsTPT1-F	ACAACATGGGCGAGGATCAT
	OsTPT1-R	CAATCTTACCACCGCAATATGC
	OsTPT2-F	TAGTTGGGTAGCTGCTTTGATCGA
	OsTPT2-R	AAATGGGATGATGGAGGCTTTG
	OsGWD-F	GCAGAAGCTGGCCAGGCAGT
	OsGWD-R	ACGTTGCGAGACTTGGCCCC
	OsISA3-F	ACAGCTTGAGACACTGGGTTGAG
	OsISA3-R	GCATCAAGAGGACAACCATCTG
	OskIF14.3-F	CATGACAAGGACATTTCAGCTCTCC
	OsKIF14.3-R	GGCAGAAAACCCTGATACTTCCTG
Subcellular localization	GFP-OsKIF14.3-F1	TCCGGAGCTAGCTCTAGAATGGTGAGCAAGGGCGAGGAGC
	GFP-OsKIF14.3-R1	CCCTCCATTGCTGCCTCTTCCATCTTGTACAGCTCGTCCATGCC
	GFP-OsKIF14.3-F2	GGCATGGACGAGCTGTACAAGATGGAAGAGGCAGCAATGGAGGG

	GFP-OsKIF14.3-R2	CAAATGTTTGAACTGCAG TGTCAGGCTAGACATCCTTCTCCTGTG
	mCherry-TUB1-F1	TCCGGAGCTAGCTCTAGAATGGTGAGCAAGGGCGAGGA
	mCherry-TUB1-R1	ATGTGCAGGATCTCCCTCATCAGCTTCGATCTCTTGTACA
	mCherry-TUB1-F2	TGTACAAGAGATCGAAGCTGATGAGGGAGATCCTGCACAT
	mCherry-TUB1-R2	CAAATGTTTGAACTGCAGCTCGTCTTCGGGGACCTGCT
	OsSUTI-mCherry-F	ACAGCCCAGATCAACTAGTATGGCTCGCGGCAGCGG
	OsSUTI-mCherry-R	CCTCGAGACGTCTCTAGAGTGACCGCCGCCCATGC
	OsSUT2-mCherry-F	ACAGCCCAGATCAACTAGTATGCCGCGGCGGCCTAG
	OsSUT2-mCherry-R	CCTCGAGACGTCTCTAGATCGGTGACCTCTCCTCCTTGATGC
	OsSWEET5-mCherry-F	ACAGCCCAGATCAACTAGTATGGTGATGAACCCTGACGCCGTCCG
	OsSWEET5-mCherry-R	CCTCGAGACGTCTCTAGACACAGCGGAGGAGTCGCCGTCCTT
	OsSWEET11-mCherry-F	ACAGCCCAGATCAACTAGTATGGCAGGAGGTTTCTTGTCCATGGC
	OsSWEET11-mCherry-R	CCTCGAGACGTCTCTAGACACGGCGGCGGTGATCTCGATCA
	OsSWEET14-mCherry-F	ACAGCCCAGATCAACTAGTATGGCTGGCATGTCTCTTCAGCATCC
	OsSWEET14-mCherry-R	CCTCGAGACGTCTCTAGATGCAGCTACCTTCTTCTCGCCGGC
Yeast two-hybrid assay	BD-CC1-F	GCCATGGAGGCCGAATTCATGGAAGAGGCAGCAATGGAGGG
	BD-CC1-R	CGCTGCAGGTCGACGGATCCCAGGTCTATGAGCTTGTCCAGTGCCTC
	AD-CC1-F	GAGGCCAGTGAATTCATGGAAGAGGCAGCAATGGAGGG
	AD-CC1-R	GAGCTCGATGGATCCCAGGTCTATGAGCTTGTCCAGTGCCTC
	BD-CC2-F	GCCATGGAGGCCGAATTC GACAT AAAGAAG CTGAAGCAGAAGAG
	BD-CC2-R	CGCTGCAGGTCGACGGATCCTGTCAGGCTAGACATCCTTCTCCTGTG
	AD-CC2-F	GAGGCCAGTGAATTCGACAT AAAGAAG CTGAAGCAGAAGAG
	AD-CC2-R	GAGCTCGATGGATCCTGTCAGGCTAGACATCCTTCTCCTGTG
BiFC	nYFP-OsKIF14.3-F	AGGACGCCGGCGGATCCATGGAAGAGGCAGCAATGGAGGG
	nYFP-OsKIF14.3-R	GAAAGCTCTGCAGGTCGACTGTCAGGCTAGACATCCTTCTCCTGTG
	cYFP-	CAGGTACCCGGGGATCCATGGAAGAGGCAGCAATGGAGGG

	OsKIF14.3-F	
	cYFP-<br>OsKIF14.3-R	CTGCCACCGCC GTCGACTGTCAGGCTAGACATCCTTCTCCTGTG
